# Rapid Blood Transfusion: The Importance of Hemodilution and Needleless Connectors

**DOI:** 10.7759/cureus.13999

**Published:** 2021-03-19

**Authors:** Mark A Burbridge, Anil K Panigrahi, Sarah A Stone, Richard A Jaffe, John Brock-Utne

**Affiliations:** 1 Anesthesiology, Stanford University Medical Center, Stanford, USA; 2 Transfusion Medicine; Anesthesiology, Stanford University Medical Center, Stanford, USA

**Keywords:** massive blood transfusion, packed red blood cell transfusion

## Abstract

Introduction: Large-bore cannulas are critical to administering IV fluids and blood products during resuscitation and treatment of hemorrhage. Although catheter flow rates for crystalloid solutions are well defined, rapid administration of blood products is poorly characterized. In this in vitro study, we examined the effects of hemodilution and needleless connectors on red blood cell (RBC) flow rates.

Methods: To determine RBC flow rates through large-bore cannulae, a crystalloid solution (Normosol®, Hospira, Lake Forest, IL) or RBC units were warmed and delivered under pressure (360 mmHg) using a Level 1 H-1200 Fast Flow Fluid Warmer (Smiths Medical, St. Paul, MN). Flow rates for crystalloid, packed RBCs and diluted RBCs were determined using a stopwatch. Additionally, the effect of the MaxPlus® clear needleless connector (CareFusion, San Diego, CA) was measured in all three infusion groups.

Results: Flow rates for undiluted RBC units were 53% slower than crystalloid solution (220 mL/min vs. 463 mL/min; p=0.0003), however, when RBC units were diluted to a hematocrit of ~30% flow rate improved to 369 mL/min (p=0.005). The addition of the MaxPlus® needleless connector reduced flow of crystalloid solution by 47% (245 mL/min; p=0.0001), undiluted RBCs by 64% (78 mL/min; p=0.01), and diluted RBCs by 51% (180 mL/min; p=0.00003). Compared to undiluted RBC units, hemodilution increased RBC delivery rate through a MaxPlus® connector by 130% (p=0.004) and by 68% (p=0.02) when the catheter was directly connected to the Level 1 tubing (MaxPlus® excluded).

Conclusion: In settings requiring rapid transfusion of RBC units, needleless connectors should not be used and hemodilution should be considered in order to decrease the time required to deliver an equivalent red cell mass.

## Introduction

Large-bore IV cannulae are critical to administering IV fluids and blood products during resuscitation and treatment of hemorrhage. Although catheter flow rates for rapid transfusion of crystalloid solutions and red blood cell (RBC) transfusions are well defined [1], strategies to safely increase the speed of RBC transfusions are lacking.

A method that may increase the speed of RBC transfusion through rapid transfusion devices is hemodilution. Hemodilution, by decreasing viscosity, will increase flow rates and may decrease the time necessary to transfuse a unit of RBC. However, it is unknown if the effect of the decrease in viscosity would offset the time necessary to deliver the expanded volume of a diluted unit of RBCs.

In addition, incorporating needleless connectors in IV tubing sets has increased in an effort to reduce catheter-related infections and to reduce exposure risk when obtaining blood samples [2-4]. However, these connectors have been shown to substantially reduce the flow of crystalloid solutions through large-bore catheters [1, 5].

In this in vitro study, we examined the effect of hemodilution on the speed of transfusion of a unit of RBC with or without the use of needleless connectors using a Level 1® rapid infusion system (Smiths Medical, St. Paul, MN).

## Materials and methods

To determine RBC flow rates through large-bore cannulae, a crystalloid solution (Normosol®, Hospira, Lake Forest, IL) or RBC units were delivered into an empty IV plastic infusion bag using a Level 1® H-1200 Fast Flow Fluid Warmer and D70 IV fluid administration set placed at a height of six feet (Smiths Medical, St. Paul, MN) connected to a 14 gage x 1.25-inch Introcan Safety IV® catheter (B Braun Medical, Bethlehem, PA). Two Adsol (AS-1) RBC units were combined to create a total volume of 500 mL with a hematocrit of 61.9% as confirmed by laboratory measurement. Flow rates for crystalloid, undiluted RBCs, and RBCs diluted with Normosol® to a hematocrit of 29.9% were measured. One liter bags of Normosol® were used because this size of bag is commonly used in clinical practice when rapid fluid administration is desired. Additionally, the effect of the MaxPlus® needleless connector (CareFusion, San Diego, CA) was measured in all three conditions. Crystalloid or RBC bags containing measured volumes were pressurized in the Level 1® chamber to a starting pressure of approximately 360 mmHg (as measured by an in-line pressure transducer placed at a side port of the Level 1 tubing to eliminate any additional resistance), hung at a height of six feet, heated to 37 degrees Celsius, and the time from the start to the end of flow was measured by a stopwatch. The same bags of undiluted and diluted RBCs were used for uniformity purposes. The same Level 1 tubing set-up was used for the entire experiment, but was thoroughly flushed when diluted and undiluted RBC bags were run. Each flow condition was tested in triplicate. Statistical analysis included ANOVA and a one-tailed t-test. A one-tailed t-test was chosen because it was not necessary to test the possibility that Normosol® would infuse slower than undiluted or diluted RBC units. RBC units were expired and donated by the Stanford University blood bank.

## Results

**Figure 1:**
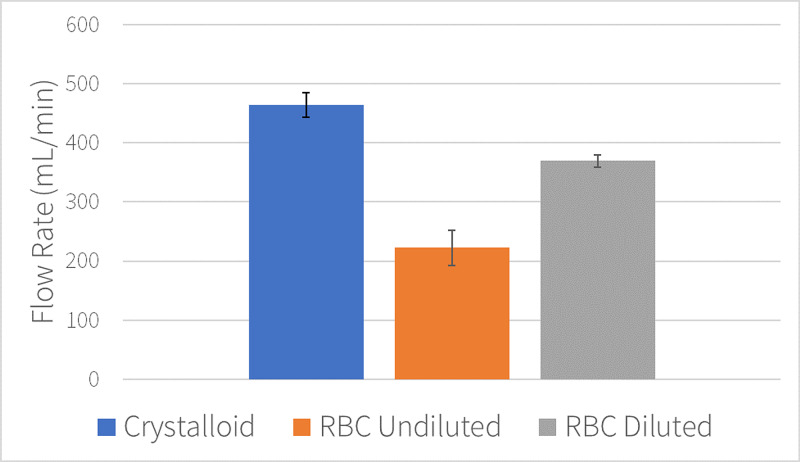
Pressurized 14 g catheter flow rates. Mean catheter flow rates with standard deviation error bars.

Mean pressurized flow rates were 463 mL/min for crystalloid (standard deviation=20.63 mL/min), 220 mL/min for undiluted RBC units (standard deviation=29.98 mL/min), and 369 mL/min for diluted RBC units (standard deviation=10.58 mL/min). Pressurized flow rates for undiluted RBC units were 53% slower than crystalloid solution (220 mL/min vs. 463 mL/min; p=0.0003; Figure 1), however, when RBC units were diluted to a hematocrit of ~30% flow rate improved to 369 mL/min (p=0.005; Figure 1).

**Figure 2:**
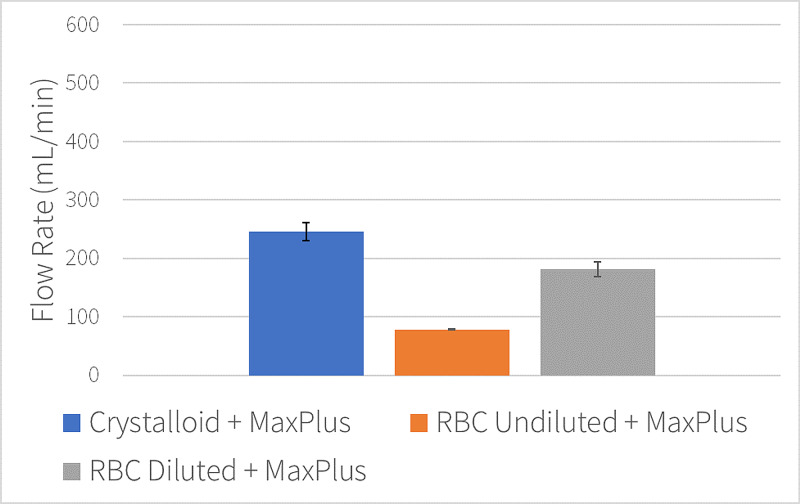
Pressurized 14 g catheter flow rates with needleless connector. Mean catheter flow rates with standard deviation error bars.

**Figure 3:**
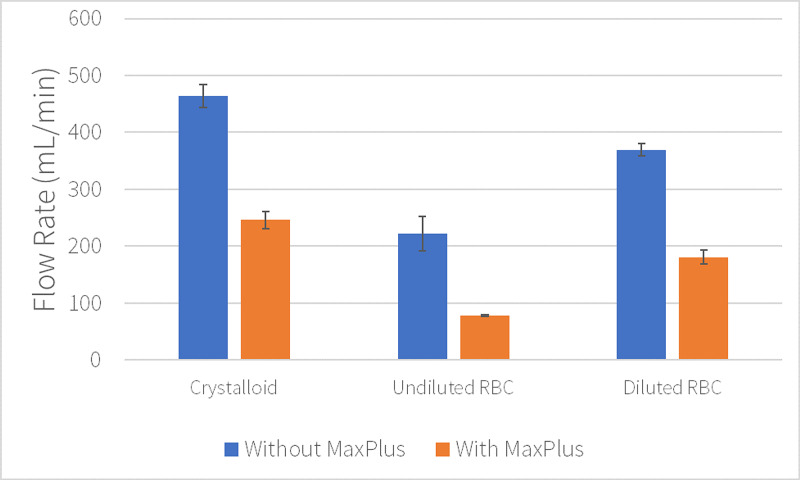
Effect of needleless connector. Mean catheter flow rates with standard deviation error bars.

The addition of the MaxPlus® needleless connector resulted in mean flow rates of 245 mL/min using crystalloid (standard deviation=14.97 mL/min), 78 mL/min using undilted RBC units, and 180 mL/min for diluted RBC units (standard deviation=12.42 mL/min). The addition of the MaxPlus® needleless connector reduced pressurized flow of crystalloid solution, undiluted RBC, and diluted RBC by 47% (245 mL/min; p=0.0001), 64% (78 mL/min; p=0.01), and 51% (180 mL/min; p=0.00003, Figures 2-3), respectively.

Compared to undiluted RBC units, hemodilution increased RBC flow rates by 130% (p=0.004) through a MaxPlus® connector (Figure 2) and up to 68% (p=0.02) when the catheter was directly connected to the Level 1 tubing (Figure 1).

**Figure 4:**
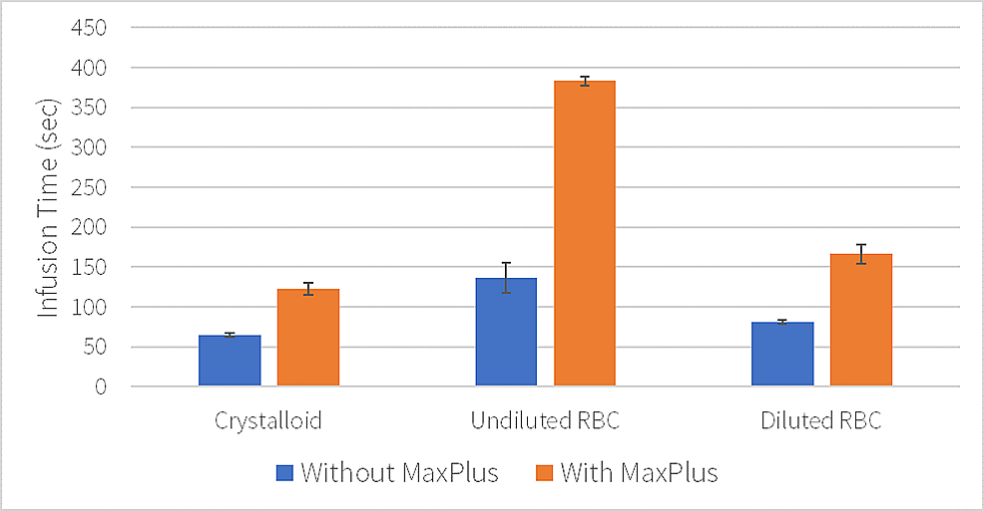
Time required to infuse 500 mL of solution. Mean catheter flow rates with standard deviation error bars.

Similarly, the time for an infusion of 500 mL of each solution was affected by the inclusion of a MaxPlus® connector (Figure 4). Mean infusion time for crystalloid and diluted RBC solutions through the needleless connector was approximately double (65-122 s, p=0.001, and 81-166 s, p=0.007, respectively), while the mean time for undiluted RBC was most greatly affected, increasing by 282% (136-383 s, p=0.002). RBC hemodilution decreased mean infusion time by 40% when the needleless connector was omitted (p=0.04).

## Discussion

Rapid administration of IV fluids including crystalloid and blood products is essential during resuscitation and/or hemorrhage. There is increased use of needleless connectors to minimize catheter-related infections and blood exposure. In this study, we question the flow difference between rapidly administering crystalloid or blood products through these connectors.

Our findings demonstrate that when using a Level 1 rapid infusion system, omitting a needleless connector can increase flow rates for undiluted RBCs by a factor of 2.86. Furthermore, our study demonstrates that when the resistance of a system is increased the relative importance of viscosity is greater.

There are many factors that can contribute to increased resistance across a system in clinical practice including patient-specific blood composition, use of extension tubing, stopcocks, drip chambers, and fluid warmers [1, 6]. Resistance to flow can be determined by Poiseuille’s Law: 𝑅=8​​​​​​*nlp*𝑟4 where *R* = resistance, *n* = viscosity, *l* = length, and *r* = radius, and any of these variables may be modified to alter resistance. Blood, a non-Newtonian fluid, can be subject to sludging and Rouleaux formation at low flow rates [7]. Additionally, a decrease in temperature can produce a significant increase in blood viscosity [8].

In order to responsibly utilize blood, our team procured two units of expired RBC (102 and 60 days past labeled expiration). Expired blood products are subject to decreased RBC deformability and increased hemolysis. While decreased RBC deformability affects flow in vivo, further experimentation is needed to determine whether this would affect flow through a large-bore IV catheter. Increased hemolysis, can cause increased blood viscosity however, we did not measure hemolysis in our RBC units [9].

## Conclusions

In conclusion, in clinical situations necessitating rapid transfusion of RBC units, needleless connectors should not be used. If needleless connectors are used, then hemodilution should be considered to decrease the time required to deliver an equivalent red cell mass.
